# Combination antiretroviral therapy prevents SIV-induced aging in the hippocampus and neurodegeneration throughout the brain

**DOI:** 10.1007/s13365-025-01275-6

**Published:** 2025-10-30

**Authors:** Miranda D. Horn, Alison R. Van Zandt, Cecily C. Midkiff, Ahmad A. Saied, Andrew G. MacLean

**Affiliations:** 1 Brain Institute, Tulane University, New Orleans, LA USA; 2 Biomedical Sciences Training Program, Tulane University School of Medicine, New Orleans, LA USA; 3 Division of Comparative Pathology, Tulane National Primate Research Center, Covington, LA USA; 4 Tulane Center for Aging, New Orleans, LA USA; 5 Louisiana Cancer Research Center, New Orleans, LA USA; 6 Tulane Center for Aging, New Orleans, LA USA; 7 Department of Microbiology and Immunology, Tulane University School of Medicine, New Orleans, LA USA; 8 Present Address: Department of Biological Sciences, Ohio Wesleyan University, Delaware, OH USA

**Keywords:** Neuropathology, Inflammaging, Senescence, Glia, Aging, HIV, Neurodegeneration

## Abstract

Virus-induced accelerated aging has been proposed as a potential mechanism underlying the persistence of HIV-associated brain injury (HABI) despite advances in access and adherence to combination antiretroviral therapies (cART). While some studies have demonstrated evidence of accelerated aging in PLWH, studies examining the effects of cART intervention are limited, with most studies being in vitro or utilizing small animal models. Here, we utilized archival FFPE tissues from Simian immunodeficiency virus (SIV) infected rhesus macaques to assess the levels of two proteins commonly associated with aging - the cellular senescence marker p16^INK4a^ (p16) and the NAD-dependent deacetylase sirtuin 1 (SIRT1). Our central hypothesis was that SIV infection induces accelerated aging phenotypes in the brain characterized by increased expression of p16 and SIRT1 that correlate with increased neurodegeneration, and that cART inhibits this process. We found that SIV infection induced increased GFAP, p16, SIRT1, and neurodegeneration in multiple brain regions. Importantly, cART reduced SIV-induced accelerated aging (p16 and SIRT1) in the hippocampus and neurodegeneration and GFAP expression in the frontal lobe. Combined, these data suggest that cART is both safe and effective in reducing neuroinflammation and age-associated alterations in astrocytes that contribute to neurodegeneration, providing possible therapeutic targets in the treatment of HABI.

**Supplementary Information:**

The online version contains supplementary material available at 10.1007/s13365-025-01275-6.

## Introduction

The advent and use of combination antiretroviral therapies (cART) has greatly improved the lifespan of people living with HIV (PLWH) to near that of the HIV-negative population. However, the onset of age-related disorders in PLWH remains over a decade earlier. Within the brain, use of cART has drastically decreased the prevalence of the most severe form of HIV-associated brain injury (HABI(Nightingale 2023)– HIV-associated dementia, yet the prevalence of milder forms – Asymptomatic Neurocognitive Impairment and Mild Neurocognitive Disorder – has increased. Additionally, whole brain imaging and neurocognitive assessments indicate an accelerated aging phenotype in PLWH even with an undetectable viral load (Petersen 2021). Whether cART contributes to neurodegeneration via direct neurotoxicity, incomplete suppression of viral replication in the CNS, or other mechanisms is yet to be determined (Lanman 2021).

An emerging theory is that chronic viral infection triggers an accelerated biological aging process. This is supported by multiple molecular markers of aging being altered throughout peripheral tissues of PLWH. For example, expression of the cellular senescence marker p16^INK4a^(p16) in T cells correlated with chronological age in uninfected controls and HIV-infected, cART-treated individuals, but was significantly higher in HIV-infected untreated individuals, indicating an accelerated aging phenotype (Nelson 2012; Ribeiro 2016). Additionally, in a longitudinal study of PLWH, epigenetic aging in peripheral blood mononuclear cells was accelerated with HIV infection and decelerated after successful viral suppression with cART (Schoepf 2023), demonstrating an accelerated aging phenotype with HIV infection that is inhibited by cART. However, when looking at what is occurring within the CNS, support for accelerated biological aging is more limited, especially in the context of cART.

One study of PLWH found accelerated aging in the occipital lobe and cerebellum relative to uninfected controls based on DNA methylation measures; however, they did not assess the impact of cART on this process (Horvath and Levine 2015). In HIV tat transgenic rats, p16 was elevated in both the frontal cortex and striatum in general, as well as in microglia specifically relative to wildtype animals (Thangaraj 2021). The same group also previously found sirtuin 1 (SIRT1), a NAD-dependent deacetylase implicated in lifespan regulation, to be decreased at both the mRNA and protein level in the frontal cortex of HIV transgenic rats relative to wildtype animals (Hu 2017). Together, these studies support the theory that HIV accelerates aging in the brain; however, as these studies were carried out in transgenic rodents, the impact of cART on the aging process could not be assessed.

The effects of cART on molecular markers of biological aging in the brain have largely been restricted to in vitro studies. In human astrocyte cultures, exposure of cells to antiretroviral drugs has been associated with numerous signs of biological aging, including decreased proliferation, and increased expression of senescence-associated β-galactosidase and the cell-cycle arrest protein p21 (Cohen 2017). As the penetrance of cART into the brain is limited, the effect of direct exposure to cART drugs may not be representative of what occurs in vivo. Thus, there is a critical need to assess the impact of cART on biological aging in the brain using in vivo models of infection that allow for long-term cART administration.

Research with nonhuman primates (NHPs) is essential to understanding the pathogenesis of HIV – through application of the highly-translational Simian immunodeficiency virus (SIV) – and has been critical to assessing the efficacy of various therapeutic strategies, including cART (Van Zandt and MacLean 2023). Additionally, due to their genetic and physiological similarities to humans, NHPs are essential for studying the aging process, especially in the brain (Didier 2016; Robillard 2016). Our lab recently found p16 increased with age in the frontal and temporal lobes and correlated with neurodegeneration in the frontal lobe and cerebellum of NHPs (Horn 2024). Additionally, SIRT1 expression increased with age in the temporal lobe and the expression of SIRT1 correlated with neurodegeneration in the frontal lobe. These, and other studies, highlight an important role for astrocytes in eugeric and pathologic aging (Robillard 2016). However, the impact of SIV infection and cART treatment on p16 and SIRT1 expression in astrocytes of the NHP brain has not previously been assessed.

The central hypothesis for this study was that exposure to SIV triggers an accelerated biological aging phenotype in the brain concomitant with increased neurodegeneration. As cART prevents productive viral replication, we expected treatment with cART to inhibit these alterations at least partially. To test these hypotheses, we utilized rhesus macaque tissues from multiple brain regions implicated in HABI and immunofluorescent staining techniques to assess the expression of p16, SIRT1, and the neurodegeneration marker FluoroJade C (FJC) as well as glial fibrillary acidic protein (GFAP) to assess the role of astrocytes.

## Materials and methods

### Ethics statement

All animals used for this study were from previous studies at the Tulane National Primate Research Center (TNPRC; Covington, LA) and were handled in accordance with the American Association for Accreditation of Laboratory Animal Care and NIH “Principles of Laboratory Animal Care”. All animal procedures were carried out by veterinarians and their staff as approved by the Institutional Animal Care and Use Committee of Tulane University. In accordance with endpoint policies, animals were humanely euthanized by veterinary staff by anesthesia with ketamine hydrochloride (10 mg/kg) followed by an overdose with sodium pentobarbital and necropsy. Tissues were collected and fixed for 48 h in 10% neutral buffered formalin with zinc modification prior to being embedded in paraffin.

### Selection of animals and tissues

This study utilized archival formalin-fixed paraffin-embedded (FFPE) tissues from 15 rhesus macaques. Animals ranged in age from 4 to 15 years of age and both males and females were included. Due to nonhuman primates being an acutely scarce resource and breeding-aged females rarely being used in studies without strong justification (Yost 2023), sexes in the groups were not balanced.

Animals were selected based on infection status with SIVmac239 or SIVmac251, treatment with a cART regimen of Tenofovir Disoproxil Fumarate (TDF) 5.1 mg/kg, Emtricitabine (FTC) 30 mg/kg, and Dolutegravir (DTG) 2.5 mg/kg, and availability of appropriate histological brain sections. Based on these criteria, we identified three animals that were naïve to SIV and cART and were healthy at the time of investigator-initiated euthanasia (Naïve), five animals that had been infected with SIV for greater than 55 days without receiving cART (Chronic SIV), and seven animals that had been infected with SIV for 77–91 days prior to the initiation of a daily cART regimen until the time of euthanasia (SIV-cART). For more detailed information on the animals used in this study, see Table 1.

Previous imaging studies have identified several brain regions that show atrophy in PLWH and SIV-infected macaques, including the frontal lobe, hippocampus, caudate, putamen, thalamus, and cerebellum (Clifford 2017; Li 2011; Mackiewicz 2019; Pfefferbaum 2014; Zhao 2019). Based on these findings, we used H&E slides to select FFPE tissue blocks that contained these brain regions for each animal whenever possible. Some animals did not have appropriate samples for each of the brain regions listed above, thus the number of tissues analyzed for each brain region varies. See Table 1 for the specific regions analyzed for each animal.

**Table 1:**
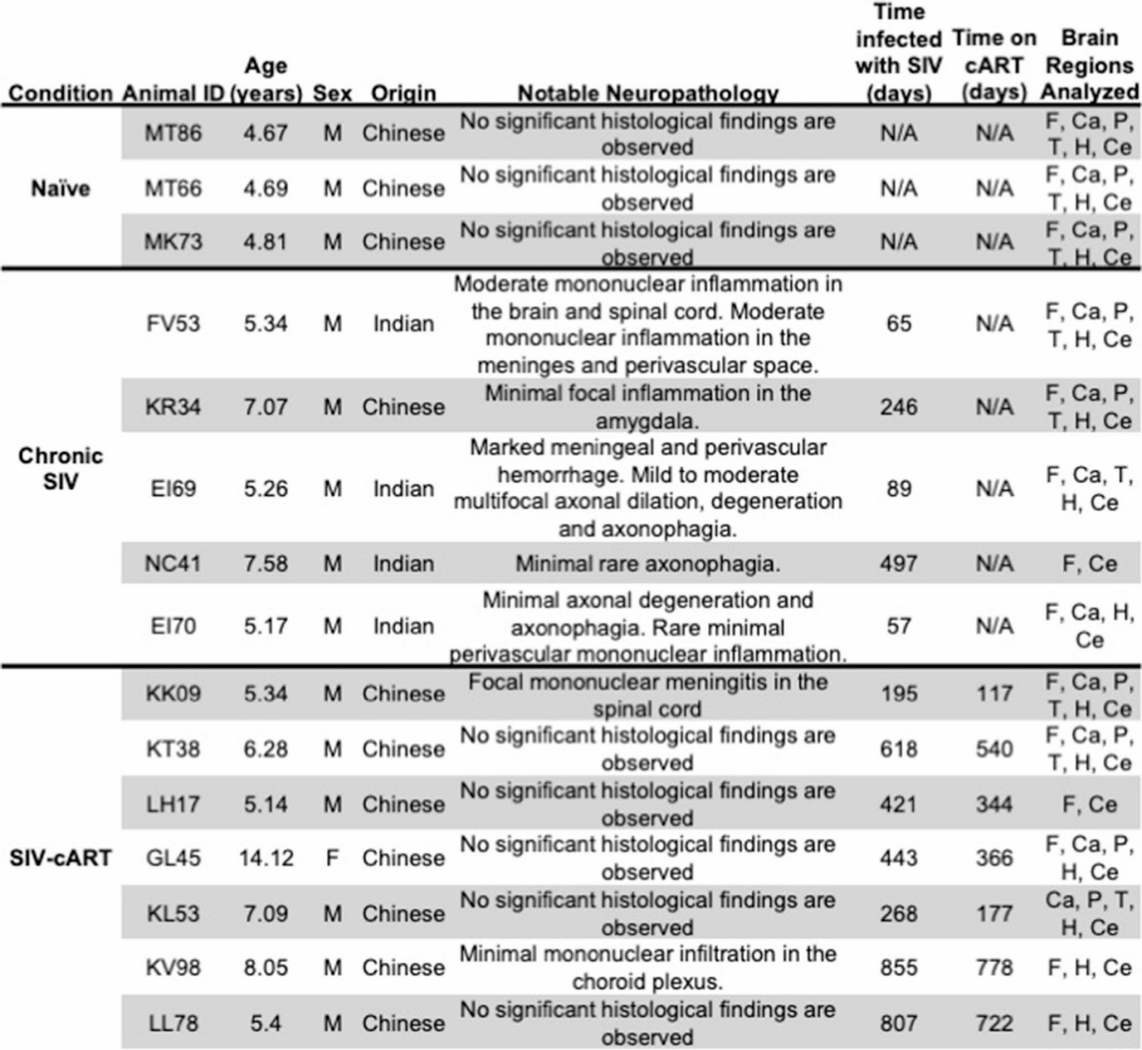
Information on the animals used in this study

Abbreviations: F = Frontal lobe, Ca = Caudate, P = Putamen, T = Thalamus, H = Hippocampus, Ce = Cerebellum.

### Immunohistochemistry

For analysis of microglia/macrophages, chromogenic immunohistochemistry for Iba1 was performed by the TNPRC Confocal Microscopy and Molecular Pathology Core. Tissues were cut at 5 μm and mounted on charged slides, then baked for at least three hours at 60 °C prior to being passed through xylene, graded ethanol, and double distilled water to remove paraffin and rehydrate tissue sections. Next, slides were boiled for 16 min in a Tris based solution, pH 9 (Vector Labs H-3301), containing 0.1% Tween20 before being briefly rinsed in hot, deionized water and transferred to a hot citrate-based solution, pH 6.0 (Vector Labs H-3300) where they were allowed to cool to room temperature. Slides were removed from the antigen retrieval solution, washed in phosphate-buffered saline, deionized water, and Roche reaction buffer before being loaded on the Ventana Discovery Ultra autostainer where they would undergo blocking, primary antibody (rabbit anti-IBA, Wako 019-19741, 1:3000 dilution) incubation, washing, secondary antibody incubation (OMap anti-Rb HRP, Ventana, cat. #760–4311), washing, DAB color development, and counterstaining with hematoxylin II. Upon removal, slides were put through alternating manual washes of deionized water containing 0.1% Dawn dish soap and plain deionized water for a total of 5 cycles. Slides were then cleared in ethanol (80%, 95%, 100%, 100%) and three xylene changes before being permanently mounted with StatLab™ AcryMount Plus Mounting Media (FisherScientific, cat. # STSL80PLUS4) and left overnight to dry prior to imaging.

### Immunofluorescent staining

For all immunohistochemistry, formalin-fixed paraffin-embedded tissues were cut at a thickness of 5 μm and placed onto charged slides. Slides were baked at 60 °C and deparaffinized by incubating three times for 5 min in xylene and rehydrating in decreasing concentrations of alcohol (100% twice for 3.5 min, 95% once for 3 min, and 80% once for 5 min). For p16^INK4a^ (anti-p16^INK4a^, Invitrogen, Ms Mab IgG1, MA5-17093, 1:100) and SIRT1 (anti-SIRT1, Lifespan Biosciences, Inc., Gt Pab, cat. #LS-B8356, 1:100), slides were rinsed in water then antigen retrieval was performed by bringing antigen-retrieval solution (Antigen Unmasking Solution, Citric Acid Based, Vector Laboratories, Inc., cat. #H-3300) to a boil, then incubating the slides in the solution uncovered for one hour. Non-specific binding was blocked by application of normal serum [normal goat (MP Biomedicals, LLC., cat. #2939149) or normal donkey (GeminiBio, cat. #100–151) serum] for 40 min prior to the application of primary antibodies. Anti-p16^INK4a^ was applied for 1 h at room temperature while anti-SIRT1 was applied overnight at 4 °C. Secondary antibodies and conjugated cell-specific antibodies (Goat anti-Mouse IgG (H + L), Alexa Fluor™ 633, Invitrogen, cat. #A-21052; Donkey anti-Goat IgG (H + L) Cross-Adsorbed Secondary Antibody, Alexa Fluor™ 568, Invitrogen, cat. #A-11057; Anti-Glial Fibrillary Acidic Protein (GFAP) − Cy3™ antibody, Sigma-Aldrich^®^, Ms Mab, cat. #C9205, 1:300; and GFAP Monoclonal Antibody (GA5), Alexa Fluor™ 488, eBioscience™, Ms Mab, cat. #53-9892-82, 1:300) were applied for 1 h at room temperature. Slides were washed twice in PBS-FSG-Tx100 (1X Phosphate-Buffered Solution diluted from 10X Phosphate-Buffered Solution, Fisher BioReagents™, cat. #BP 3994; 0.2% Fish-Skin Gelatin, Sigma Aldrich, cat. #1002923460; 0.1% Triton-100, Fisher BioReagents™, cat. #BP 151 500) for 5 min and once in 1X PBS-FSG for 5 min after incubation with each antibody. Slides were washed for 5 min in 1X PBS prior to applying mounting media (EverBrite TrueBlack^®^ Hardset Mounting Medium, Biotium, cat. #23018) and coverslips.

### FluoroJade C staining

For FJC (Fluoro-Jade C Staining Kit with DAPI Counter Stain, Histo-Chem Inc., cat. #FJC-SK-DAPI) staining, following the deparaffinization described above, slides were incubated in 70% alcohol for 2 min followed by deionized water for 2 min. Autofluorescence was dampened by incubating slides in potassium permanganate for 5 min. Slides were then washed twice in deionized water for 2 min and placed in the FJC and DAPI solution for 10 min. Slides were then washed three times in deionized water for 1 min and dried in the oven for 15 min at 60 °C. Finally, slides were cleared in xylene for 1 min prior to application of mounting media (Micromount mounting media, Leica Biosystems, cat. #3801730) and coverslips. After applying coverslips, slides were allowed to set at room temperature overnight before scanning.

### Image analysis

For GFAP, p16, and SIRT1 analyses, slides were scanned using a Zeiss AxioScan.Z1 slide scanner at 20x magnification. Scan settings were optimized for each brain region and stain and the same scan profile was used across all slides for a given brain region and stain. Scanned images were loaded into the HALO^®^ Image Analysis software (Indica Labs) for analysis. Images were annotated for appropriate regions of interest and then parameters were set using the Highplex FL module (Indica Labs) to identify phenotypes of interest. Finally, whole-slide images and regions of interest were analyzed. Data were exported to Microsoft Excel where the percentage of marker-positive cells, dual-labeled cells, and relative fluorescent intensity for each marker were extracted and organized for statistical analysis. For Iba1 and microglia activation analyses, slides were scanned using a Hamamatsu NanoZoomer360 at 40x magnification. Images were loaded into the HALO^®^ image analysis software and analyzed using optimized settings in the Microglia Activation Module for each brain region to determine the percentage of Iba1 + cells and activated Iba1 + cells.

### Statistical analyses

To assess the significance of differences in marker expression between groups, the percentage of marker-positive cells, dual-labeled cells, and relative intensity for each group were compared using a one-way ANOVA and Tukey’s post-hoc test in Prism GraphPad (version 10, GraphPad Software, La Jolla, CA). To determine the relationship between expression of multiple markers, two-tailed Pearson’s correlation coefficients were calculated using Prism GraphPad. Finally, to determine if p16 and SIRT1 expression predict neurodegeneration, separate multiple linear regressions were performed with %FJC + cells as the dependent variable or FJC intensity as the dependent variable. A p value < 0.05 was considered significant for all analyses.

## Results

### Description of pathology from selected animals

**Fig. 1:**
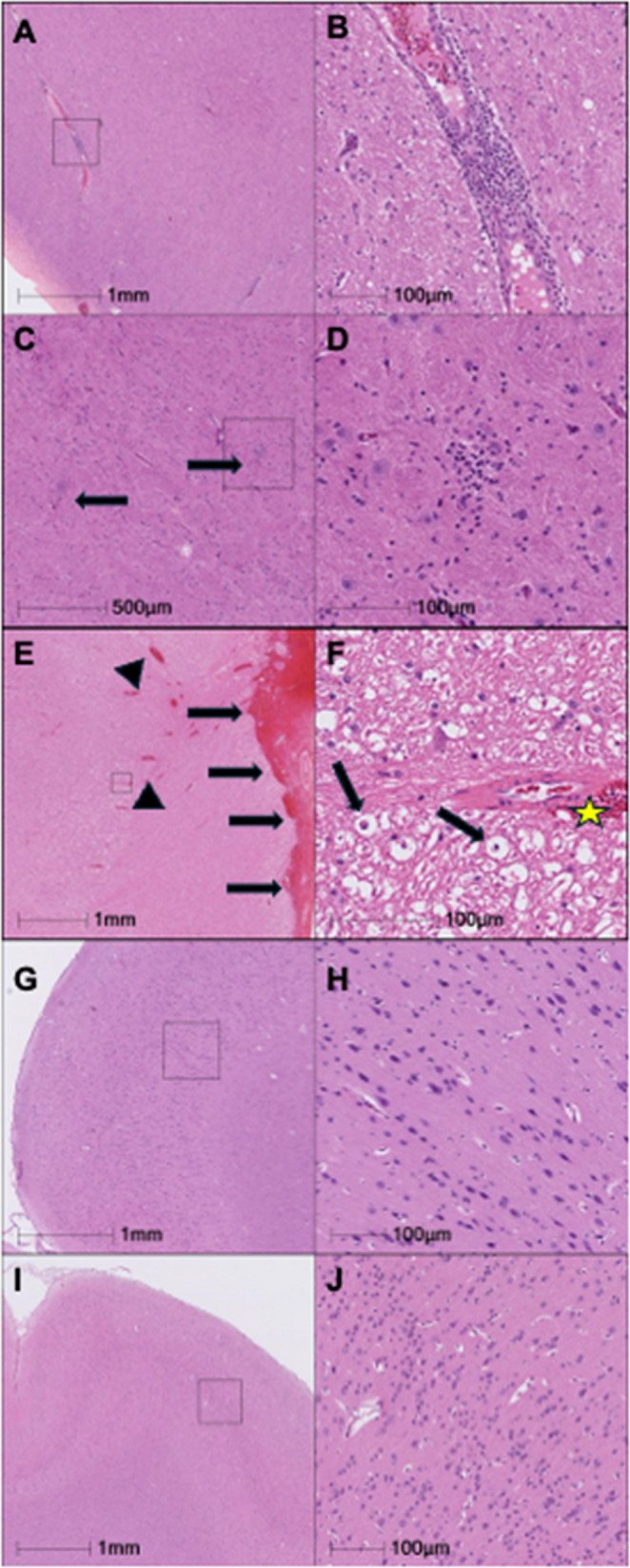
Representative histopathology of the brain. (A-D): Images from FV53 (Chronic SIV). **A** Virchow-Robin spaces are infiltrated by moderate numbers of lymphocytes and plasma cells. **B** Higher magnification of the reference frame from panel A showing perivascular inflammation. **C** Thalamus, multifocal microglial nodules (black arrows). **D** Higher magnification of the microglial nodule from the reference frame of panel C. (E-F): Images from EI69 (Chronic SIV). **E** The meninges (black arrows) and Virchow-Robin spaces (arrow heads) are markedly expanded by hemorrhage. **F** Higher magnification of the reference frame from panel E showing hemorrhage in a Virchow-Robin space (yellow star), and axonal degeneration and axonophagia (black arrows). Images from Temporal Lobes of SIV-cART group –(GL45, G & I), and Naïve group (MK73). No significant histological changes are observed (20X objective magnification

Animals infected with SIV in this study developed perivascular inflammation, meningitis, and microglial nodules in the brain with changes in the brains of chronically infected animals more pronounced than that of those treated with cART. Detailed histological examination revealed moderate inflammation in the chronically infected without cART (Table 1), and minor inflammation in the cART treated SIV-infected animals (Fig. 1). Consistent with these findings, the percentage of Iba1 + cells were elevated in chronically infected animals compared to SIV-cART animals in multiple brain regions (Supplemental Fig. 1).

### GFAP expression increases with SIV infection are region-specific

**Fig. 2:**
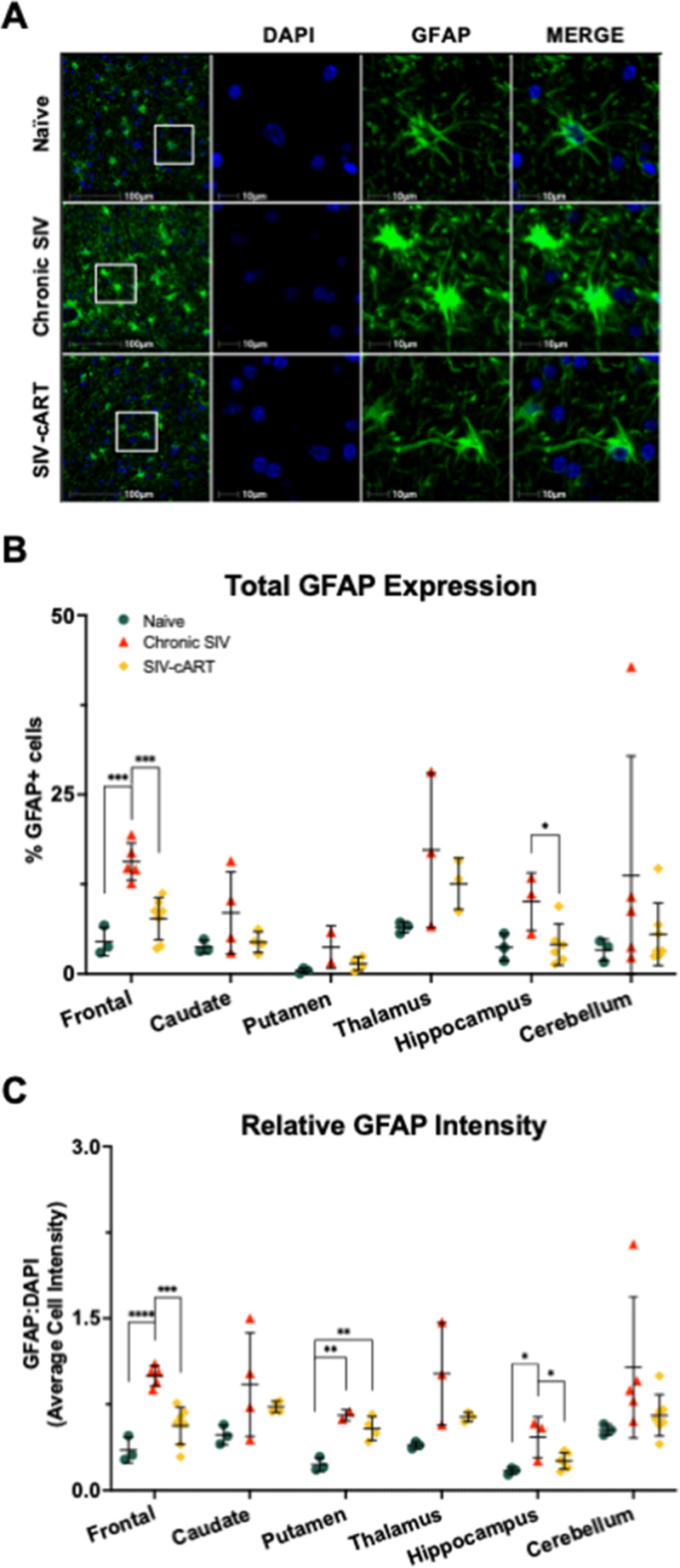
Treatment with cART reduces SIV-induced elevation of GFAP + cells in multiple brain regions of rhesus macaques. **A** Representative immunofluorescent images of GFAP staining in the white matter of the frontal lobe. **B** The percentage of GFAP + cells was significantly elevated in the frontal lobe of chronic SIV-infected animals relative to naïve and cART treated animals. **C** The average cell intensity of GFAP staining relative to that of DAPI staining was also significantly increased in the frontal lobe and hippocampus of chronic SIV-infected animals relative to naïve and cART treated animals. All data points are presented and mean +/- SD are plotted for each group. Each brain region was analyzed independently using a one-way ANOVA and Tukey’s post-hoc test. * *p* < 0.05, ** *p* < 0.01, *** *p* < 0.001, **** *p* < 0.0001

We assessed changes in astrocyte activation across all six brain regions by staining slides for DAPI (blue) and GFAP (green) and quantifying the percentage of co-labeled cells (Fig. 2). Relative to naïve animals, the percentage of GFAP + cells increased with chronic SIV infection in the frontal lobe (Fig. 2b, *p* = 0.0003). However, changes in the percentage of GFAP + cells with SIV infection did not reach statistical significance in any other brain region.

Recently, we showed that increased GFAP intensity better correlated with increasing age in rhesus macaques than the percentage of GFAP+ cells (Horn 2024). Therefore, we hypothesized that if SIV infection increases aging it would increase GFAP intensity as well. When assessing GFAP intensity relative to DAPI intensity, we found GFAP to increase with chronic SIV infection in the frontal lobe, putamen, and hippocampus (Fig.2c, *p* < 0.0001, *p* = 0.0031, and *p* = 0.0147, respectively). This data suggests that astrocytes in these regions may be more sensitive to infection with SIV or more directly impacted than astrocytes in the caudate, thalamus, and cerebellum.

To our knowledge, the effect of long-term cART on GFAP expression across multiple brain regions has not previously been assessed in vivo. Here, we show that treatment with cART significantly decreased both the percentage of GFAP + cells and relative intensity of GFAP compared with that of chronically SIV-infected animals in the frontal lobe (Fig. 2b, *p* = 0.0007; Fig. 2c, *p* = 0.0003) and hippocampus (*p* = 0.0479 and *p* = 0.0418, respectively). Importantly, the only place we observed a significant increase in GFAP intensity in cART-treated animals relative to naïve animals was in the putamen (Fig. 2c, *p* = 0.0061). Overall, it appears as though treatment with cART reduces GFAP expression in SIV-infected animals and thus likely decreases inflammation in the brain.

### p16INK4a expression increases with SIV infection across all brain regions

As p16 increases with age in rhesus macaques, especially in the frontal lobe (Horn 2024), we hypothesized that SIV would increase p16 expression throughout the selected brain regions indicative of premature or accelerated aging. To test this, we stained slides from each brain region for DAPI, GFAP, and p16 (red), obtained whole-slide images, and assessed the percentage of p16 + cells in the total cell population and in the GFAP + cell population, as well as the relative intensity of p16.

**Fig. 3:**
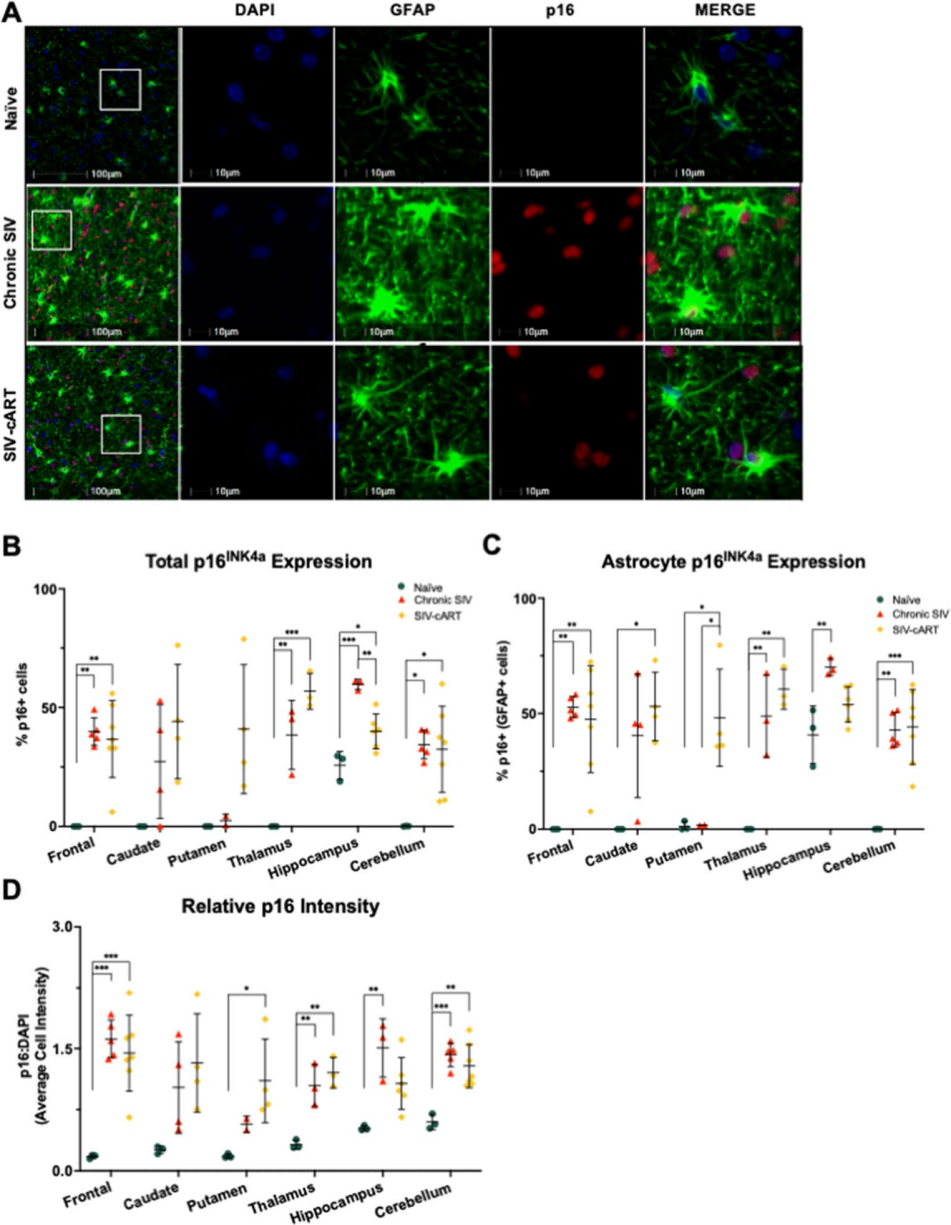
p16^INK4a^ expression is elevated across multiple brain regions in SIV-infected rhesus macaques. **A** Representative immunofluorescent images of p16 staining across groups in the white matter of the frontal lobe. **B** The percentage of p16 + cells is elevated in SIV-infected animals relative to naïve animals in all brain regions except the caudate and putamen. **C** The percentage of p16 + astrocytes is elevated in chronic SIV-infected animals relative to naïve animals in all brain regions except the caudate and putamen where only SIV-cART animals showed elevated p16 + astrocytes. **D** The relative intensity of p16 staining is also elevated in SIV-infected animals relative to naïve animals in all brain regions except the caudate and putamen. All data points are presented and mean +/- SD are plotted for each group. Each brain region was analyzed independently using a one-way ANOVA and Tukey’s post-hoc test. * *p* < 0.05, ** *p* < 0.01, *** *p* < 0.001

Relative to young, naïve rhesus macaques (4–5 years of age), where there was minimal expression of the senescence marker, p16 expression was robustly increased across the frontal lobe, thalamus, hippocampus, and cerebellum in chronic SIV infection (Fig. 3). The total percentage of p16 + cells was increased in the frontal lobe, thalamus, hippocampus, and cerebellum relative to naïve animals (Fig. 3b, *p* = 0.0017, *p* = 0.0060, *p* = 0.0002, and *p* = 0.0104, respectively). The percentage of p16 + cells was also significantly elevated in the frontal lobe, thalamus, hippocampus, and cerebellum of cART-treated animals relative to naïve animals (*p* = 0.0020, *p* = 0.0008, *p* = 0.0241, and *p* = 0.0107, respectively). It is important to note that only in the hippocampus did treatment with cART significantly reduce the percentage of p16 + cells from that in chronically SIV-infected animals (Fig. 3b, *p* = 0.0037). This suggests that while cART was effective in reducing productive viral infection peripherally, it did not significantly alter the expression of p16 following chronic SIV-infection in the brain.

We observed a similar pattern for the percentage of p16 + astrocytes (Fig. 3c) with increases in the frontal lobe, thalamus, hippocampus, and cerebellum of chronically infected animals relative to naïve animals (*p* = 0.0024, *p* = 0.0047, *p* = 0.0052, and *p* = 0.0012, respectively). The percentage of p16 + astrocytes was also elevated in cART-treated animals relative to naïve animals across the frontal lobe, caudate, putamen, thalamus, and cerebellum (*p* = 0.0034, *p* = 0.0148, *p* = 0.0148, *p* = 0.0016, and *p* = 0.0006, respectively) and relative to chronically infected animals in the putamen (*p* = 0.0267). Thus, outside the effect in the putamen, cART does not significantly alter the expression of p16 in the brain from that seen in chronically infected animals.

To assess changes in the overall expression of p16, we analyzed the intensity of p16 staining relative to that of DAPI and again saw a similar pattern of increased p16 expression with SIV infection across most regions of the brain (Fig. 3d). With chronic infection, we saw a significant increase in p16 expression in the frontal lobe, thalamus, hippocampus, and cerebellum (*p* = 0.0004, *p* = 0.0074, *p* = 0.0063, *p* = 0.0004, respectively). We also saw a significant increase in p16 expression in cART-treated animals relative to naïve animals in the frontal lobe, putamen, thalamus, and cerebellum (*p* = 0.0007, *p* = 0.0378, *p* = 0. 0028, *p* = 0.0013, respectively), with no significant reductions relative to SIV-infected, untreated animals. Thus, cART does not appear to alter overall p16 expression from that seen in untreated, SIV-infected animals.

### SIRT1 expression varies based on brain region and infection status

SIRT1 expression correlates with neurodegeneration in the frontal lobe of uninfected animals (Horn 2024). Additionally, it is known that the activity of SIRT1 is inhibited by the HIV protein Tat leading to hyperactivation of immune cells (Kwon 2008). Thus, we were interested in how these interactions may impact expression in the brain, especially in astrocytes. Based on the reduction of SIRT1 at the mRNA and protein levels in the gut of SIV-infected animals (Mohan 2015), we hypothesized there would be similar reductions in the brain. To test this hypothesis, we obtained whole-slide scanned images and assessed the percentage of SIRT1 + cells, the percentage of SIRT1 + astrocytes, and the relative intensity of SIRT1 staining.

In general, the relationship between SIV infection and SIRT1 expression was highly region specific. In the cerebellum, we saw a significant increase in the percentage of SIRT1 + cells with chronic infection, relative to naïve animals (*p* = 0.0087). In the frontal lobe, caudate, putamen, thalamus, and hippocampus we did not see any significant changes in the percentage of SIRT1 + cells following chronic infection. However, cART had opposing effects based on brain region examined. In the frontal lobe and putamen, we saw a significant increase in SIRT1 + cells in cART-treated animals relative to naïve animals (*p* = 0.0140 and *p* = 0.0388, respectively). Whereas in the hippocampus, cART-treated animals had a significant reduction in SIRT1 + cells relative to both naïve and chronically infected animals (*p* = 0.0002 and *p* < 0.0001, respectively).

**Fig. 4:**
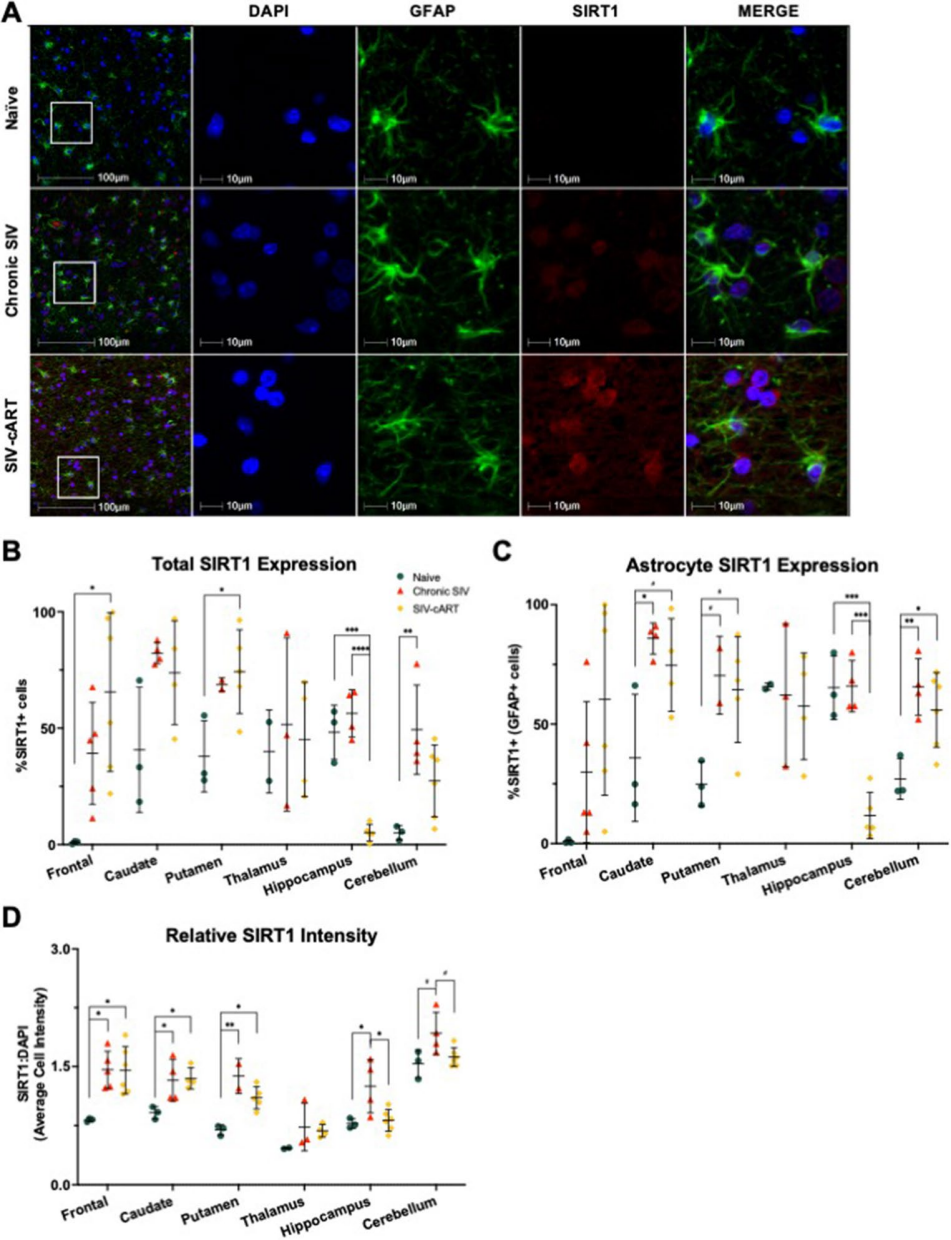
Expression of SIRT1 varies with SIV infection in several brain regions of the rhesus macaque. **A** Representative immunofluorescent images of SIRT1 staining across groups in the white matter of the frontal lobe. **B** Relative to naïve animals, the percentage of SIRT1 + cells was elevated in the frontal lobe and putamen of cART-treated animals and cerebellum of chronic SIV animals but reduced in the hippocampus of cART-treated animals. **C** Relative to naïve animals, the percentage of SIRT1 + astrocytes was elevated in the caudate of chronic SIV animals and in the cerebellum of chronic SIV animals and cART-treated animals but reduced in the hippocampus of cART-treated animals. **D** The average cell intensity of SIRT1 staining relative to DAPI was elevated in the frontal lobe, caudate, putamen, and hippocampus of chronic SIV animals and in the frontal lobe, caudate, and putamen of cART-treated animals. In the hippocampus, cART-treated animals had significantly reduced SIRT1 staining relative to chronic, untreated animals. All data points are presented and mean +/- SD are plotted for each group. Each brain region was analyzed independently using a one-way ANOVA and Tukey’s post-hoc test. ^#^ *p* < 0.1, * *p* < 0.05, ** *p* < 0.01, *** *p* < 0.001, **** *p* < 0.0001

Similar to the total cell population, there were region-specific changes in the percentage of SIRT1 + astrocytes (Fig. 4c). We saw a significant increase in SIRT1 + astrocytes in chronically infected animals relative to naïve animals in the caudate (*p* = 0.0177) and cerebellum (*p* = 0.0093), with a similar trend in the putamen (*p* = 0.0703). Additionally, cART-treated animals had elevated SIRT1 + astrocytes in the cerebellum relative to naïve animals (*p* = 0.0303) with a similar trend in the caudate (*p* = 0.0559) and putamen (*p* = 0.0514). However, cART significantly reduced SIRT1 + astrocytes in the hippocampus relative to both naïve animals (*p* = 0.0002) and chronically infected animals (*p* = 0.0001).

We also analyzed the intensity of SIRT1 staining relative to DAPI staining (Fig. 4d). Here, we saw more consistent patterns across the brain. Chronically infected animals had a significant increase in SIRT1 expression relative to naïve animals in the frontal lobe (*p* = 0.0128), caudate (*p* = 0.0477), putamen (*p* = 0.0026), and hippocampus (*p* = 0.0366). Treatment with cART did not significantly reduce the relative SIRT1 expression in the frontal lobe, caudate, or putamen, where it was still significantly elevated relative to naïve animals (*p* = 0.0111, *p* = 0.0371, and *p* = 0.0125, respectively). In the hippocampus, SIRT1 expression was significantly reduced in cART-treated animals relative to chronically infected animals (*p* = 0.0246) to levels that resemble that seen in naïve animals. A similar trend was seen in the cerebellum, where SIRT1 intensity was elevated in chronically infected animals and then reduced with cART treatment, though it did not reach statistical significance (*p* = 0.0501 and *p* = 0.0713, respectively).

### FluoroJade C staining is increased in chronically SIV-infected animals in a region-specific manner

**Fig. 5:**
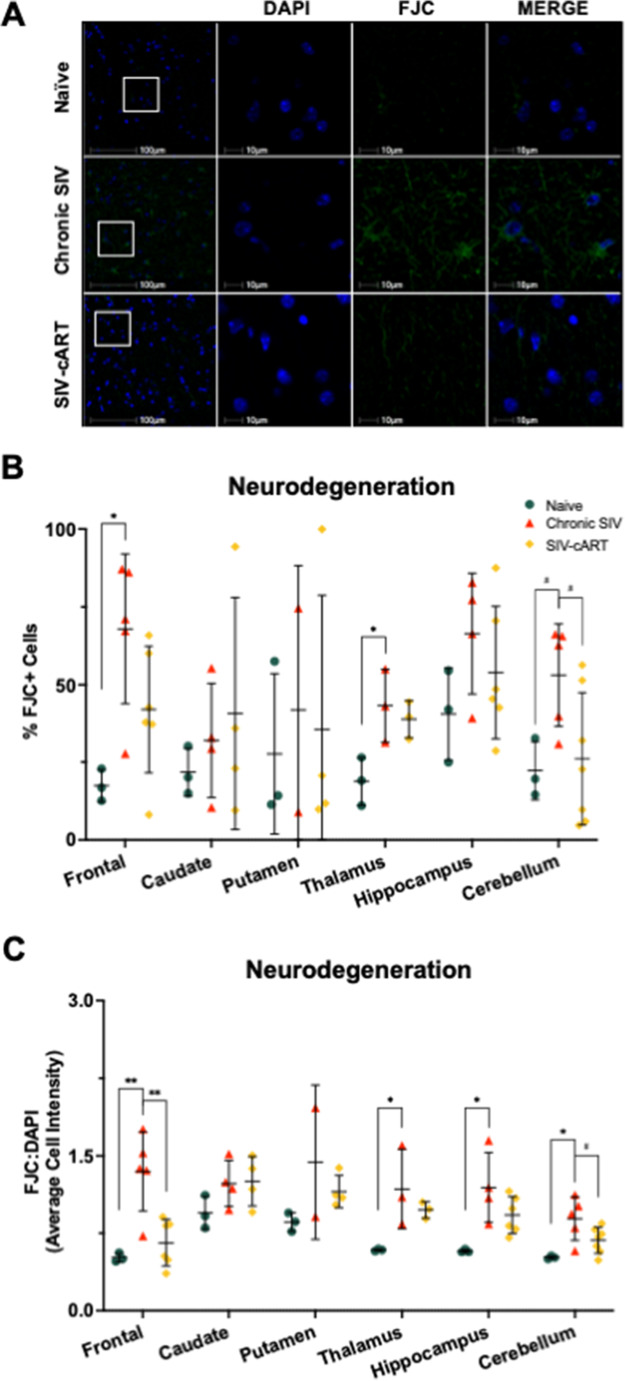
Untreated chronic SIV infection induces neurodegeneration in multiple brain regions of the rhesus macaque. **A** Representative immunofluorescent images of FJC staining across groups in the white matter of the frontal lobe. **B** The percentage of FJC + cells is elevated in the frontal lobe, thalamus, and cerebellum of chronic SIV animals. **C** The average cell intensity of FJC staining relative to DAPI staining is elevated in the frontal lobe, thalamus, hippocampus, and cerebellum of chronic SIV animals. Treatment with cART significantly reduces FJC staining in the frontal lobe from that seen in chronic SIV animals. All data points are presented and mean +/- SD are plotted for each group. Each brain region was analyzed independently using a one-way ANOVA and Tukey’s post-hoc test. ^#^ *p* < 0.1, * *p* < 0.05, ** *p* < 0.01

Neurodegeneration was increased, as measured by the percentage of FJC + cells or relative intensity of FJC staining, in chronically SIV-infected animals across several brain regions relative to naïve animals, but not to cART treated animals (Fig. 5). There was a significant increase in the percentage of FJC + cells (Fig. 5b), relative to naïve animals in the frontal lobe (*p* = 0.0142) and thalamus (*p* = 0.0346), with the cerebellum showing a similar trend (*p* = 0.0926). When looking at the relative intensity of FJC staining, we saw a similar pattern (Fig. 5c). Relative FJC intensity increased with chronic infection relative to naïve animals again in the frontal lobe (*p* = 0.0044) thalamus (*p* = 0.0438), hippocampus (*p* = 0.0122), and cerebellum (*p* = 0.0128). Interestingly, when using relative intensity of FJC as a measure of general neurodegeneration, cART-treated animals had significantly less neurodegeneration in the frontal lobe than chronically infected animals (*p* = 0.0045) with a similar trend in the cerebellum (*p* = 0.0834), suggesting a potential protective effect of cART. It is important to note that neurodegeneration was not significantly elevated in cART-treated animals relative to naïve animals in any brain region by either measure used, and therefore does not appear to be eliciting additional neurodegeneration in the brain.

### Neurodegeneration correlates with markers of accelerated aging

**Fig. 6:**
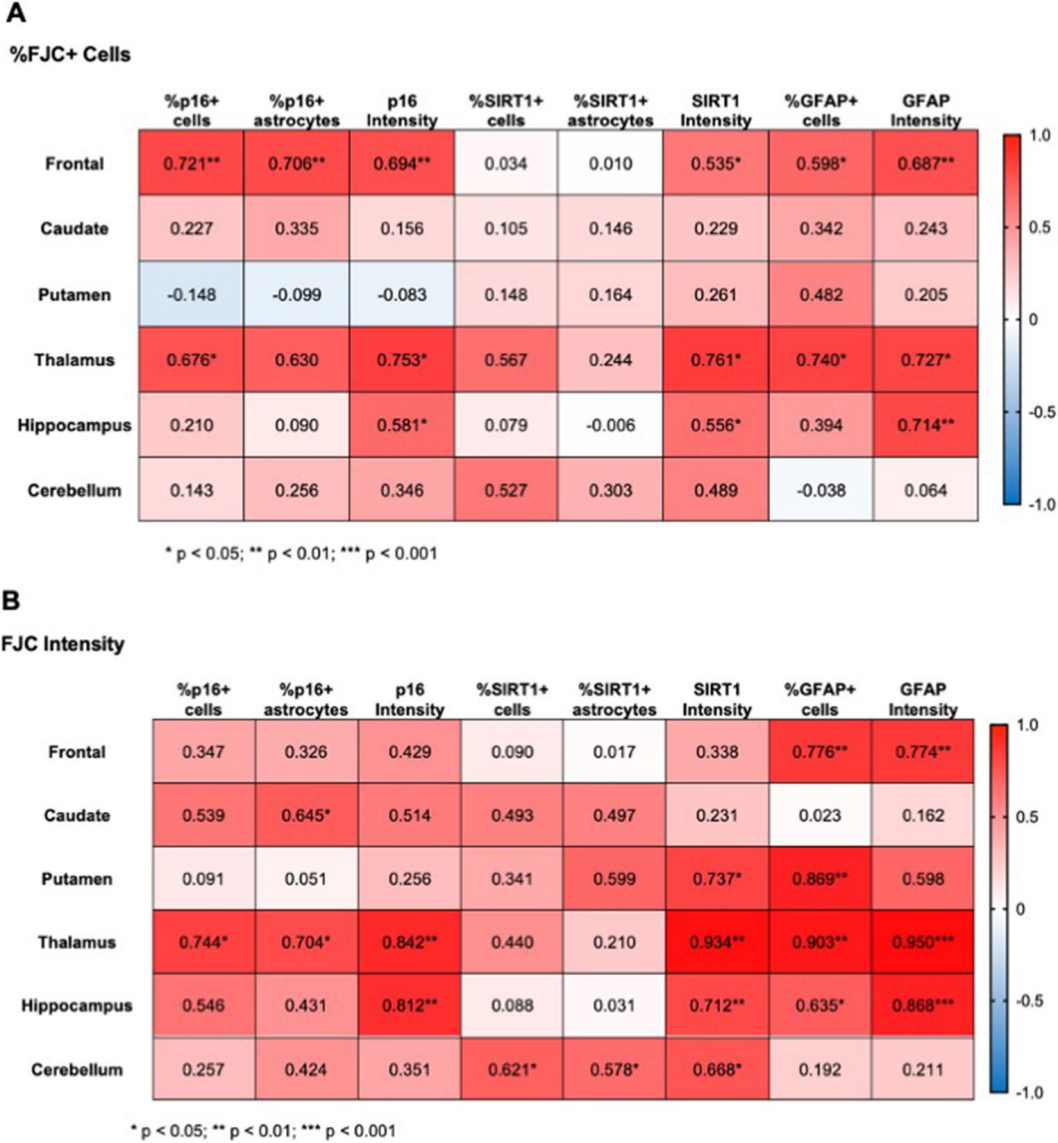
Expression of aging markers and GFAP positively correlate with neurodegeneration. **A** Correlations between aging markers, GFAP, and the percentage of FJC + cells. **B** Correlations between aging markers, GFAP, and the average cell intensity of FJC staining relative to DAPI staining. Values presented are Pearson’s correlation coefficients with two-tailed *p* values. * *p* < 0.05, ** *p* < 0.01, *** *p* < 0.001

Finally, to assess the relevance of changes in p16 and SIRT1 expression to neurodegeneration, we performed Pearson correlation analyses between each marker and neurodegeneration (Fig. 6). Here we found a great deal of variability from one brain region to the next and depending on the measure of neurodegeneration used. When assessing the correlation of aging markers with the percentage of FJC + cells (Fig. 6a), the %p16 + cells were significantly correlated in the frontal lobe (*p* = 0.004) and thalamus (*p* = 0.046) while the %p16 + astrocytes were only significantly correlated in the frontal lobe (*p* = 0.005). However, p16 intensity correlated with %FJC + cells in the frontal lobe (*p* = 0.006), thalamus (*p* = 0.019), and hippocampus (*p* = 0.047). The %SIRT1 + cells and the %SIRT1 + astrocytes did not correlate with %FJC + cells in any brain region. Yet, SIRT1 intensity correlated with %FJC + cells in the frontal lobe (*p* = 0.049), thalamus (*p* = 0.028), and hippocampus (*p* = 0.049). Finally, the %GFAP + cells correlated with %FJC + cells in both the frontal lobe (*p* = 0.024) and thalamus (*p* = 0.026), and the intensity of GFAP staining correlated with %FJC + cells in the frontal lobe (*p* = 0.007), thalamus (*p* = 0.026), and hippocampus (*p* = 0.009).

When assessing the correlation of aging markers with the intensity of FJC staining (Fig. 6b), the %p16 + cells and p16 intensity were both significantly correlated in the thalamus (*p* = 0.022 and *p* = 0.004, respectively) while p16 intensity was correlated in the hippocampus (*p* = 0.001). The %p16 + astrocytes was also significantly correlated with FJC intensity in the caudate (*p* = 0.032) and thalamus (*p* = 0.034). The %SIRT1 + cells and %SIRT1 + astrocytes was only correlated with FJC intensity in the cerebellum (*p* = 0.024 and *p* = 0.038, respectively). However, the intensity of SIRT1 staining correlated with FJC intensity in several brain regions, including the putamen (*p* = 0.024), thalamus (*p* = 0.001), hippocampus (*p* = 0.006), and cerebellum (*p* = 0.012). Finally, the %GFAP + cells and GFAP intensity both correlated with FJC intensity in the frontal lobe (*p* = 0.001 and *p* = 0.001, respectively), thalamus (*p* = 0.001 and *p* = 0.00008789, respectively), and hippocampus (*p* = 0.020 and *p* = 0.0002481, respectively), while only %GFAP + cells correlated with FJC intensity in the putamen (*p* = 0.002).

To determine if p16 and SIRT1 significantly predicted neurodegeneration, multiple linear regression was used. Here, we found both p16 expression and SIRT1 expression to predict FJC expression, but only in the frontal lobe and hippocampus (Supplemental Fig. 2). For the frontal lobe, when assessing if %p16 + cells and %SIRT1 + cells predicted %FJC + cells, the overall regression was significant (R^2^ = 0.6395, F(3, 10) = 5.912, *p* = 0.0138) and the %p16 + cells significantly predicted %FJC + cells (ß = 1.347, *p* = 0.0046). For the frontal lobe, when assessing if %p16 + astrocytes and %SIRT1 + astrocytes predicted %FJC + cells, the overall regression was significant (R^2^ = 0.5803, F(3, 10) = 4.609, *p* = 0.0284) and the %p16 + astrocytes significantly predicted %FJC + cells (ß = 0.8968, *p* = 0.0096). In the hippocampus, %p16 + cells significantly predicted %FJC + cells (ß = −2.506, *p* = 0.0400), as did the %SIRT1 + cells (ß = −2.710, *p* = 0.0201), and the interaction between %p16 + cells and %SIRT1 + cells (ß = 0.06263, *p* = 0.0180). Similarly for the hippocampus, when assessing if %p16 + astrocytes and %SIRT1 + astrocytes predicted %FJC + cells, the overall regression was significant (R^2^ = 0.7842, F(3, 8) = 9.689, *p* = 0.0049) and the %p16 + astrocytes significantly predicted %FJC + cells (ß = −3.634, *p* = 0.0013), as did the %SIRT1 + astrocytes (ß = −3.923, *p* = 0.0007), and the interaction between %p16 + astrocytes and %SIRT1 + astrocytes (ß = 0.07095, *p* = 0.0007). We saw similar results in the hippocampus when assessing if %p16 + cells and %SIRT1 + cells predicted FJC intensity, where the overall regression was significant (R^2^ = 0.6960, F(3, 8) = 6.105, *p* = 0.0183) and the %SIRT1 + cells significantly predicted FJC intensity (ß = −0.03755, *p* = 0.0122), as did the interaction between %p16 + cells and %SIRT1 + cells (ß = 0.0008458, *p* = 0.0123). Similarly for the hippocampus, when assessing if %p16 + astrocytes and %SIRT1 + astrocytes predicted FJC intensity, the overall regression was significant (R^2^ = 0.6356, F(3, 8) = 4.652, *p* = 0.0365) and the %SIRT1 + astrocytes significantly predicted FJC intensity (ß = −0.04555, *p* = 0.01457), as did the interaction between %p16 + astrocytes and %SIRT1 + astrocytes (ß = 0.0008220, *p* = 0.0139). Together, these results demonstrate the importance of p16 and SIRT1 in SIV-induced neurodegeneration in the frontal lobe and hippocampus, especially in astrocytes.

## Discussion

In this study, utilizing archival rhesus macaque tissues from six brain regions implicated in HABI, we demonstrated significant alterations of the senescence/aging markers p16 and SIRT1 in both the total cell population and in astrocytes with SIV infection that correlated with neurodegeneration. The astrocyte marker GFAP increased with infection in several brain regions but was decreased with cART treatment, suggestive of a protective effect of cART (Fig. 2). Expression of p16 was significantly elevated with SIV infection across most brain regions for both the total cell population and astrocytes specifically (Fig. 3). Similarly, expression of SIRT1 was elevated with SIV infection in several brain regions (Fig. 4). Importantly, cART treatment only led to significant decreases in p16 and SIRT1 expression relative to chronic, untreated infection in the hippocampus, with significant increases relative to naïve animals in several brain regions (Figs. 4 and 5). Additionally, neurodegeneration significantly increased in chronically infected, untreated animals relative to naïve animals across several brain regions and treatment with cART significantly decreased neurodegeneration relative to chronically infected animals in the frontal lobe (Fig. 5). Furthermore, the expression of p16 and SIRT1 correlated with neurodegeneration in both the total cell population and in astrocytes across several brain regions (Fig. 6). Together, these data provide critical new insights into SIV-induced, aging-related changes throughout the brain.

While GFAP expression is widely used as a marker of astrocyte reactivity or astrogliosis, it has also been shown to increase with age. In a recent study of eugeric aging in the rhesus macaque brain, we found that while the percentage of GFAP + cells did not correlate with age, the intensity of GFAP staining was correlated with age in multiple brain regions (Horn 2024). Consistent with these findings, expression of GFAP has also been shown to increase with age across multiple regions of the brain in both rodents and humans (Nichols 1993; O’Callaghan and Miller 1991). Additionally, the size and morphology of astrocytes have been shown to change with age in humans and NHPs (Robillard 2016; Jyothi 2015; Kanaan et al. 2010), consistent with an increase in GFAP expression but not necessarily an increase in the number of GFAP + cells. This is of particular interest here as the intensity of GFAP, but not the percentage of GFAP + cells, increased with SIV infection in the putamen and hippocampus. Combined with the results for p16 and SIRT1, these results indicate a potential sensitivity of these regions to the aging effects of chronic infection with SIV.

As one of the most prominent markers of cellular senescence, p16 has been associated with aging in many species and cell types. Within the brain, p16 has been shown to increase with normal aging in the frontal cortex of humans (Idda 2020)and in astrocytes specifically (Bhat 2011). We found p16 to increase with normal aging in astrocytes of the frontal and temporal lobes of rhesus macaques but not in the parietal lobe or cerebellum (Horn 2024). Thus, p16 likely increases with age in a region-specific manner. In the context of HIV, p16 has been shown to increase in the frontal lobe of PLWH and in microglia of HIV tat-transgenic rats (Thangaraj 2021). Within astrocytes, in vitro exposure to HIV proteins increases expression of p16 (Pillai 2023; Yu 2017). Similarly, cortical astrocytes in a humanized HIV mouse model have elevated levels of p16 compared to control animals (Yu 2017). However, astrocyte expression of p16 throughout the brain of SIV-infected NHPs had not been previously analyzed, especially in the context of suppressive cART. Here, we demonstrate robust increases in astrocytic p16 with SIV infection across brain regions implicated in HABI. These increases are also seen in the total cell population, which warrants further investigation to determine if other cell types experience similar significant increases in p16 expression with SIV infection or if this is an astrocyte-driven response. Importantly, astrocyte expression of p16 could be indicative of cellular senescence in these cells which would prevent them from performing critical homeostatic functions and lead to damaging effects in the CNS. However, as p16 expression alone is not sufficient to determine a cellular senescence phenotype these studies assessed the expression of additional molecular markers associated with aging to better assess the status of these cells.

Another molecular marker associated with aging and an upstream regulator of the cellular senescence marker p21, is SIRT1, an NAD-dependent deacetylase. SIRT1 acts largely in an anti-aging capacity, as it inhibits several pathways associated with aging and is downregulated with age in several tissues (Xu 2020). However, the downregulation of SIRT1 expression is not consistent across all tissues, suggesting a tissue-specific alteration with age. Within the brain, SIRT1 expression has been shown to increase with age across several brain regions of Wistar rats while it is the activity of SIRT1 that is decreased (Braidy 2015). We also observed an increase in SIRT1 expression with age in the temporal lobe of NHPs (Horn 2024), but have not yet assessed SIRT1 activity. In the context of HIV, SIRT1 expression is decreased in the gut of SIV-infected rhesus macaques (Mohan 2015)and in macrophages/microglia of NHPs with SIV-encephalitis (Chaudhuri 2013). As the HIV-1 Tat protein is known to directly interact with SIRT1 and inhibit its activity (Kwon 2008), a decrease in expression combined with a decrease in activity could lead to drastic increases in aging processes. However, in the present study, we found both total SIRT1 expression and astrocytic SIRT1 expression to be increased with SIV infection across several brain regions (Fig. 4). It is possible these increases in expression are combined with decreases in activity as seen with normal aging in Wistar rats (Braidy 2015) and may even represent a compensatory mechanism to counteract the inhibition in activity brought on by Tat.

While cART treatment has been shown to reduce molecular markers of aging from that seen in chronically infected, untreated individuals in peripheral blood (Nelson 2012; Ribeiro 2016; Schoepf 2023), to our knowledge the impact of cART on biological aging across multiple brain regions has not previously been reported. Here, we found that p16 levels in cART treated animals only differed from that seen in chronically SIV-infected, untreated animals in a couple of brain regions. In the hippocampus, we observed significant decreases in the %p16 + cells amongst cART treated animals relative to chronically infected animals (Fig.3b). However, these levels of p16 were still significantly higher than that seen in naïve animals, suggesting that initiation of cART may be able to slow the aging process in the hippocampus but not return it to normal. Further studies are needed to determine if this accelerated aging is the result of chronic infection, or if acceleration is initiated during very early infection events.

Conversely, we saw an increase in the %p16 + astrocytes in cART treated animals compared to chronically infected, untreated animals in the putamen, but this may be due to a limited sample size for putamen from chronic SIV animals and warrants further investigation. For SIRT1, we saw a similar effect in the hippocampus, with expression levels being significantly reduced from that seen in chronically infected, untreated animals (Fig. 4). Together, these data suggest that cART may be important for limiting SIV-induced aging in the hippocampus which could have important implications for PLWH. It is also important to note that neurodegeneration in cART-treated animals was not significantly different from naïve animals in any brain region and was reduced from that seen in chronically infected, untreated animals in the frontal lobe, suggesting cART is protective against SIV-induced neurodegeneration.

These findings may hold clinical relevance for PLWH. Human imaging studies on brain aging in PLWH have demonstrated premature, accentuated, or accelerated aging throughout regions of the brain implicated by the cognitive and motor deficits seen in HABI, including the frontal cortex, caudate, putamen, hippocampus, thalamus, and cerebellum (Clifford 2017; Pfefferbaum 2014; Ances 2012; Boban 2018; Cardenas 2009; Cole 2017; Cysique 2013; Davies 2019; Harezlak 2011; Lew 2021; Nir 2021; Seider 2016; Towgood 2012). Importantly, cognitive decline also shows premature onset or accelerated progression in PLWH (Goodkin 2017; Sheppard 2017)and post-mortem analyses demonstrate age-related epigenetic alterations in CNS tissues (Horvath and Levine 2015; Gross 2016; Sehl 2020). While we did not conduct brain imaging or cognitive assessments on the animals in this study, previous studies in non-human primates have demonstrated the clinical relevance of SIV-infected monkeys. For example, studies using imaging techniques similar to those used in humans have also identified brain alterations in SIV infected rhesus macaques across brain regions implicated in HABI (Li 2011; Zhao 2019; Fuller 2004; Gonzalez 2018; Ratai 2009; Schreiber-Stainthorp 2018; Song 2020; Tang 2016; Wu 2013).

## Conclusion

Overall, this study demonstrates for the first time that multiple markers of aging are significantly elevated, especially in astrocytes, with SIV infection across several brain regions implicated in HABI. Additionally, these alterations are not only correlated with neurodegeneration but are also predictive of neurodegeneration in the frontal lobe and hippocampus suggesting a causative role. Given the importance of these brain regions for learning/memory and executive functioning, two cognitive domains that have been shown to be significantly more impaired in PLWH in the cART era than pre-cART (Heaton 2011), our results may have critical implications in the treatment or prevention of HAND. More work is needed to determine the direct effects of these aging markers on the progression of neurodegeneration and development of HAND and the underlying mechanisms at play.

## Supplementary Information

Below is the link to the electronic supplementary material.

Supplementary Material 1Supplemental Fig.1: SIV-induced increases in microglia/macrophages are reduced with cART. (A) Representative images of immunohistochemistry for Iba1, with the middle panel showing a magnification of the boxed area in the left panel, and the right panel depicting the microglia activation results. Green is homeostatic or non-activated and red is amoeboid/activated with black lines representing the total process area. (B) The percentage of Iba1 + cells in cART-treated animals is significantly reduced in the caudate, putamen, and hippocampus. (C) The percentage of activated Iba1 + cells is also decreased in the caudate relative to SIV-infected, untreated animals and is significantly reduced in the thalamus in all SIV-infected animals relative to naïve animals. All data points are presented and mean +/- SD are plotted for each group. Each brain region was analyzed independently using a one-way ANOVA and Tukey’s post-hoc test. * *p* < 0.05, ** *p* < 0.01.

Supplementary Material 2Supplemental Fig.2: Pearson correlation coefficients between all markers for each brain region

## Data Availability

No datasets were generated or analysed during the current study.

## References

[CR1] Ances BM et al (2012) Independent effects of HIV, aging, and HAART on brain volumetric measures. J Acquir Immune Defic Syndr 59(5):469–477 22269799 10.1097/QAI.0b013e318249db17 PMC3302928

[CR2] Bhat R et al (2011) Human astrocytes exhibit an increased expression of the cellular senescence biomarker p16INK4a in aged human brain and alzheimer’s disease. FASEB J 25(S1):p6153–p6153

[CR3] Boban JM et al (2018) Early introduction of cART reverses brain aging pattern in Well-Controlled HIV infection: A comparative MR spectroscopy study. Front Aging Neurosci 10:329 30405398 10.3389/fnagi.2018.00329 PMC6200868

[CR4] Braidy N et al (2015) Differential expression of sirtuins in the aging rat brain. Front Cell Neurosci 9:167 26005404 10.3389/fncel.2015.00167 PMC4424846

[CR5] Cardenas VA et al (2009) Evidence for ongoing brain injury in human immunodeficiency virus-positive patients treated with antiretroviral therapy. J Neurovirol 15(4):324–333 19499454 10.1080/13550280902973960 PMC2889153

[CR6] Chaudhuri AD et al (2013) Up-regulation of microRNA-142 in Simian immunodeficiency virus encephalitis leads to repression of sirtuin1. FASEB J 27(9):3720–3729 23752207 10.1096/fj.13-232678 PMC3752547

[CR7] Clifford KM et al (2017) Progressive brain atrophy despite persistent viral suppression in HIV patients older than 60 years. J Acquir Immune Defic Syndr 76(3):289–297 28650401 10.1097/QAI.0000000000001489 PMC5634906

[CR8] Cohen J et al (2017) Astrocyte senescence and metabolic changes in response to HIV antiretroviral therapy drugs. Front Aging Neurosci 9:281 28900395 10.3389/fnagi.2017.00281 PMC5581874

[CR9] Cole JH et al (2017) Increased brain-predicted aging in treated HIV disease. Neurology 88(14):1349–1357 28258081 10.1212/WNL.0000000000003790 PMC5379929

[CR10] Cysique LA et al (2013) HIV, vascular and aging injuries in the brain of clinically stable HIV-infected adults: a (1)H MRS study. PLoS ONE 8(4):e61738 23620788 10.1371/journal.pone.0061738 PMC3631163

[CR11] Davies O et al (2019) Clinical and neuroimaging correlates of cognition in HIV. J Neurovirol 25(6):754–764 31214916 10.1007/s13365-019-00763-w PMC6920239

[CR12] Didier ES et al (2016) Contributions of nonhuman primates to research on aging. Vet Pathol 53(2):277–290 26869153 10.1177/0300985815622974 PMC5027759

[CR13] Fuller RA et al (2004) A prospective longitudinal in vivo 1H MR spectroscopy study of the siv/macaque model of neuroaids. BMC Neurosci 5:10 15070430 10.1186/1471-2202-5-10 PMC385227

[CR14] Gonzalez RG et al (2018) Temporal/compartmental changes in viral RNA and neuronal injury in a primate model of neuroaids. PLoS ONE 13(5):e0196949 29750804 10.1371/journal.pone.0196949 PMC5947913

[CR15] Goodkin K et al (2017) Effect of ageing on neurocognitive function by stage of HIV infection: evidence from the multicenter AIDS cohort study. Lancet HIV 4(9):e411–e422 28716545 10.1016/S2352-3018(17)30098-X PMC5753579

[CR16] Gross AM et al (2016) Methylome-wide analysis of chronic HIV infection reveals Five-Year increase in biological age and epigenetic targeting of HLA. Mol Cell 62(2):157–168 27105112 10.1016/j.molcel.2016.03.019 PMC4995115

[CR17] Harezlak J et al (2011) Persistence of HIV-associated cognitive impairment, inflammation, and neuronal injury in era of highly active antiretroviral treatment. AIDS 25(5):625–633 21297425 10.1097/QAD.0b013e3283427da7 PMC4326227

[CR18] Heaton RK et al (2011) HIV-associated neurocognitive disorders before and during the era of combination antiretroviral therapy: differences in rates, nature, and predictors. J Neurovirol 17(1):3–16 21174240 10.1007/s13365-010-0006-1 PMC3032197

[CR19] Horn MD et al (2024) Astrocyte expression of aging-associated markers positively correlates with neurodegeneration in the frontal lobe of the rhesus macaque brain. Front Aging Neurosci 16:1368517 38577492 10.3389/fnagi.2024.1368517 PMC10993697

[CR20] Horvath S, Levine AJ (2015) HIV-1 infection accelerates age according to the epigenetic clock. J Infect Dis 212(10):1563–1573 25969563 10.1093/infdis/jiv277 PMC4621253

[CR21] Hu G et al (2017) Tat-Mediated induction of miRs-34a & -138 promotes astrocytic activation via downregulation of SIRT1: implications for aging in HAND. J Neuroimmune Pharmacol 12(3):420–432 28236278 10.1007/s11481-017-9730-0 PMC5546000

[CR22] Idda ML et al (2020) Survey of senescent cell markers with age in human tissues. Aging 12(5):4052–4066 32160592 10.18632/aging.102903 PMC7093180

[CR23] Jyothi HJ et al (2015) Aging causes morphological alterations in astrocytes and microglia in human substantia Nigra Pars compacta. Neurobiol Aging 36(12):3321–3333 26433682 10.1016/j.neurobiolaging.2015.08.024

[CR24] Kanaan NM, Kordower JH, Collier TJ (2010) Age-related changes in glial cells of dopamine midbrain subregions in rhesus monkeys. Neurobiol Aging 31(6):937–952 18715678 10.1016/j.neurobiolaging.2008.07.006 PMC2872507

[CR25] Kwon HS et al (2008) Human immunodeficiency virus type 1 Tat protein inhibits the SIRT1 deacetylase and induces T cell hyperactivation. Cell Host Microbe 3(3):158–167 18329615 10.1016/j.chom.2008.02.002 PMC2680745

[CR26] Lanman T et al (2021) CNS neurotoxicity of antiretrovirals. J Neuroimmune Pharmacol 16(1):130–143 31823251 10.1007/s11481-019-09886-7 PMC7282963

[CR27] Lew BJ et al (2021) Reductions in Gray matter linked to epigenetic HIV-Associated accelerated aging. Cereb Cortex 31(8):3752–3763 33822880 10.1093/cercor/bhab045 PMC8258439

[CR28] Li C et al (2011) Longitudinal diffusion tensor imaging and perfusion MRI investigation in a macaque model of neuro-AIDS: a preliminary study. NeuroImage 58(1):286–292 21658455 10.1016/j.neuroimage.2011.05.068 PMC3144312

[CR29] Mackiewicz MM et al (2019) Pathogenesis of age-related HIV neurodegeneration. J Neurovirol 25(5):622–633 30790184 10.1007/s13365-019-00728-z PMC6703984

[CR30] Mohan M et al (2015) Dysregulated miR-34a-SIRT1-acetyl p65 axis is a potential mediator of immune activation in the colon during chronic Simian immunodeficiency virus infection of rhesus macaques. J Immunol 194(1):291–306 25452565 10.4049/jimmunol.1401447 PMC4272908

[CR31] Nelson JA et al (2012) Expression of p16(INK4a) as a biomarker of T-cell aging in HIV-infected patients prior to and during antiretroviral therapy. Aging Cell 11(5):916–918 22738669 10.1111/j.1474-9726.2012.00856.x PMC3697001

[CR32] Nichols NR et al (1993) GFAP mRNA increases with age in rat and human brain. Neurobiol Aging 14(5):421–429 8247224 10.1016/0197-4580(93)90100-p

[CR33] Nightingale S et al (2023) Cognitive impairment in people living with HIV: consensus recommendations for a new approach. Nat Rev Neurol 19(7):424–433 37311873 10.1038/s41582-023-00813-2

[CR34] Nir TM et al (2021) Association of immunosuppression and viral load with subcortical brain volume in an international sample of people living with HIV. JAMA Netw Open 4(1):e2031190 33449093 10.1001/jamanetworkopen.2020.31190 PMC7811179

[CR35] O’Callaghan JP, Miller DB (1991) The concentration of glial fibrillary acidic protein increases with age in the mouse and rat brain. Neurobiol Aging 12(2):171–174 1904995 10.1016/0197-4580(91)90057-q

[CR36] Petersen KJ et al (2021) Accelerated brain aging and cerebral blood flow reduction in persons with human immunodeficiency virus. Clin Infect Dis 73(10):1813–1821 33621317 10.1093/cid/ciab169 PMC8599198

[CR37] Pfefferbaum A et al (2014) Accelerated aging of selective brain structures in human immunodeficiency virus infection: a controlled, longitudinal magnetic resonance imaging study. Neurobiol Aging 35(7):1755–1768 24508219 10.1016/j.neurobiolaging.2014.01.008 PMC3980003

[CR38] Pillai PP et al (2023) Involvement of LncRNA TUG1 in HIV-1 Tat-Induced astrocyte senescence. Int J Mol Sci 24(5). 10.3390/ijms24054330 10.3390/ijms24054330 PMC10002460 36901763

[CR39] Ratai EM et al (2009) In vivo proton magnetic resonance spectroscopy reveals region specific metabolic responses to SIV infection in the macaque brain. BMC Neurosci 10:63 19545432 10.1186/1471-2202-10-63 PMC2711091

[CR40] Ribeiro SP et al (2016) p16INK4a expression and immunologic aging in chronic HIV infection. PLoS One 11(11):e0166759 27861555 10.1371/journal.pone.0166759 PMC5115792

[CR41] Robillard KN et al (2016) Glial cell morphological and density changes through the lifespan of rhesus macaques. Brain Behav Immun 55:60–69 26851132 10.1016/j.bbi.2016.01.006 PMC4899176

[CR42] Schoepf IC et al (2023) Epigenetic ageing accelerates before antiretroviral therapy and decelerates after viral suppression in people with HIV in switzerland: a longitudinal study over 17 years. Lancet Healthy Longev 4(5):e211–e218 37148893 10.1016/S2666-7568(23)00037-5

[CR43] Schreiber-Stainthorp W et al (2018) Brain (18)F-FDG PET of SIV-infected macaques after treatment interruption or initiation. J Neuroinflammation 15(1):207 30007411 10.1186/s12974-018-1244-z PMC6046092

[CR44] Sehl ME et al (2020) The effects of Anti-retroviral therapy on epigenetic age acceleration observed in HIV-1-infected adults. Pathog Immun 5(1):291–311 33501399 10.20411/pai.v5i1.376 PMC7815056

[CR45] Seider TR et al (2016) Age exacerbates HIV-associated white matter abnormalities. J Neurovirol 22(2):201–212 26446690 10.1007/s13365-015-0386-3 PMC4783252

[CR46] Sheppard DP et al (2017) Accelerated and accentuated neurocognitive aging in HIV infection. J Neurovirol 23(3):492–500 28321696 10.1007/s13365-017-0523-2 PMC5441968

[CR47] Song B et al (2020) A longitudinal study of brain volume changes in rhesus macaque model infected with SIV. J Neurovirol 26(4):581–589 32583233 10.1007/s13365-020-00864-x

[CR48] Tang Z et al (2016) Longitudinal assessment of fractional anisotropy alterations caused by Simian immunodeficiency virus infection: a preliminary diffusion tensor imaging study. J Neurovirol 22(2):231–239 26438160 10.1007/s13365-015-0388-1

[CR49] Thangaraj A et al (2021) HIV TAT-mediated microglial senescence: role of SIRT3-dependent mitochondrial oxidative stress. Redox Biol 40:101843 33385630 10.1016/j.redox.2020.101843 PMC7779826

[CR50] Towgood KJ et al (2012) Mapping the brain in younger and older asymptomatic HIV-1 men: frontal volume changes in the absence of other cortical or diffusion tensor abnormalities. Cortex 48(2):230–241 21481856 10.1016/j.cortex.2011.03.006

[CR51] Van Zandt AR, MacLean AG (2023) Advances in HIV therapeutics and cure strategies: findings obtained through non-human primate studies. J Neurovirol 29(4):389–399 37635184 10.1007/s13365-023-01162-y PMC11636591

[CR52] Wu WE et al (2013) Global Gray and white matter metabolic changes after Simian immunodeficiency virus infection in CD8-depleted rhesus macaques: proton MRS imaging at 3 T. NMR Biomed 26(4):480–488 23418159 10.1002/nbm.2889 PMC3784644

[CR53] Xu C et al (2020) SIRT1 is downregulated by autophagy in senescence and ageing. Nat Cell Biol 22(10):1170–1179 32989246 10.1038/s41556-020-00579-5 PMC7805578

[CR54] Yost OC, Downey A, Ramos KS (eds) (2023) Nonhuman primate models in biomedical research: state of the science and future needs. Washington (DC), Editors 37184189

[CR55] Yu C et al (2017) HIV and drug abuse mediate astrocyte senescence in a beta-catenin-dependent manner leading to neuronal toxicity. Aging Cell 16(5):956–965 28612507 10.1111/acel.12593 PMC5595688

[CR56] Zhao J et al (2019) Low-frequency fluctuation characteristics in rhesus macaques with SIV infection: a resting-state fMRI study. J Neurovirol 25(2):141–149 30478797 10.1007/s13365-018-0694-5

