# Supplementary figures and images for: Combination antiretroviral therapy prevents SIV-induced aging in the hippocampus and neurodegeneration throughout the brain
Source: J Neurovirol. 2025 Oct 30;31(6):485–500. doi: 10.1007/s13365-025-01275-6 (PMC12700946; doi:10.1007/s13365-025-01275-6)

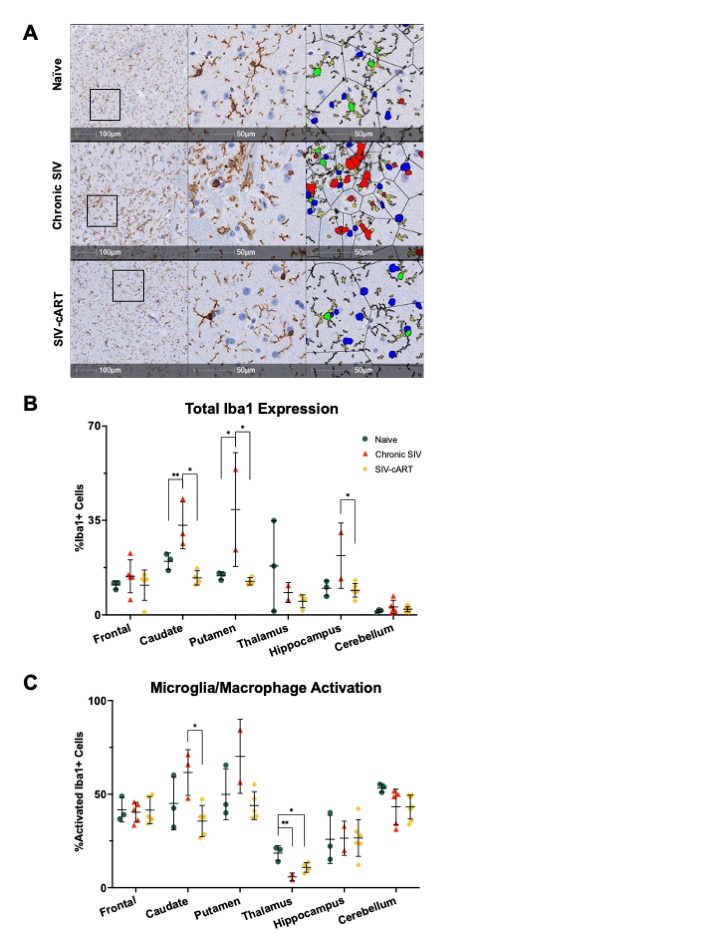

Supplement: Supplementary file 1 — Supplementary Material 1Supplemental Fig.1: SIV-induced increases in microglia/macrophages are reduced with cART. (A) Representative images of immunohistochemistry for Iba1, with the middle panel showing a magnification of the boxed area in the left panel, and the right panel depicting the microglia activation results. Green is homeostatic or non-activated and red is amoeboid/activated with black lines representing the total process area. (B) The percentage of Iba1 + cells in cART-treated animals is significantly reduced in the caudate, putamen, and hippocampus. (C) The percentage of activated Iba1 + cells is also decreased in the caudate relative to SIV-infected, untreated animals and is significantly reduced in the thalamus in all SIV-infected animals relative to naïve animals. All data points are presented and mean +/- SD are plotted for each group. Each brain region was analyzed independently using a one-way ANOVA and Tukey’s post-hoc test. * p < 0.05, ** p < 0.01. [file 13365_2025_1275_MOESM1_ESM.jpeg]

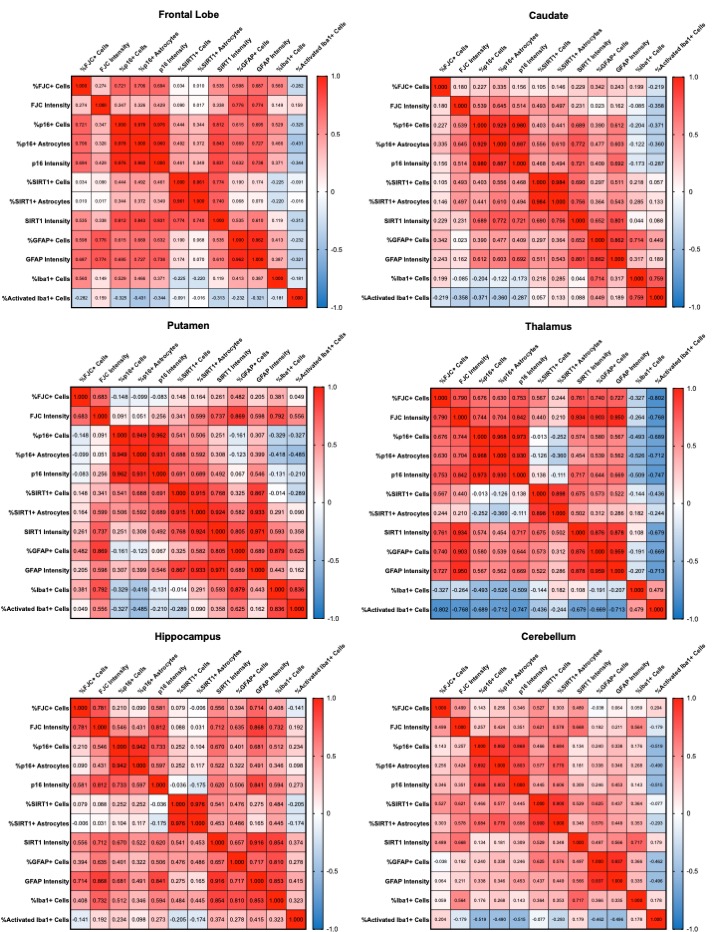

Supplement: Supplementary file 2 — Supplementary Material 2Supplemental Fig.2: Pearson correlation coefficients between all markers for each brain region [file 13365_2025_1275_MOESM2_ESM.jpeg]
